# Mimicking the Complexity of Solid Tumors: How Spheroids Could Advance Cancer Preclinical Transformative Approaches

**DOI:** 10.3390/cancers17071161

**Published:** 2025-03-30

**Authors:** Sylvia Mangani, Spyros Kremmydas, Nikos K. Karamanos

**Affiliations:** Biochemistry, Biochemical Analysis & Matrix Pathobiology Research Group, Laboratory of Biochemistry, Department of Chemistry, University of Patras, 26504 Patras, Greece

**Keywords:** solid tumors, 3D cell culture models, spheroids, matrix-independent models

## Abstract

Traditional 2D cell culture models present significant limitations in replicating the intricate architecture and microenvironment of in vivo solid tumors, which are essential for accurately studying cancer initiation, growth, progression, and metastasis. This underscores the need for the development of advanced preclinical models to accelerate research outcomes. Emerging 3D cell culture systems, particularly spheroid models, provide a more realistic representation of solid tumor properties by capturing the complex interactions occurring within the tumor microenvironment, including the extracellular matrix dynamics that influence cancer progression. Among solid tumors, breast cancer remains the most frequently diagnosed cancer among women globally and a leading cause of cancer-related mortality. Here we emphasize the value of breast cancer cell-derived spheroids in revealing differential molecular characteristics and understanding cancer cell properties during the early stages of invasion into adjacent tissues. Conclusively, this study underscores the urgent need to adopt 3D cell culture platforms, given their significant contributions to advanced cancer research and pharmaceutical targeting. This may well offer a transformative approach for preclinical studies and enhance our ability to test therapeutic efficiency in conditions that closely mimic the growth and progression of in vivo solid tumors.

## Simple Summary

Conventional 2D cell cultures cannot mimic the complex architecture and microenvironment of in vivo solid tumors, limiting their effectiveness, particularly in cancer research. Emerging 3D culture systems, especially spheroid models, offer a more accurate representation of tumor behavior by capturing dynamic microenvironmental interactions. We highlight the importance of adopting 3D culture platforms to improve preclinical cancer research, enabling more precise drug evaluation and advancing therapeutic strategies to closely resemble real tumor growth and dissemination properties.

## 1. Introduction

One of the biggest challenges of modern cancer research is the establishment of reliable in vitro experimental models that closely resemble the complexity of the tumor microenvironment (ΤΜΕ). Cell cultures are frequently utilized to understand the basic biology and mechanisms of action behind various diseases. Traditional two-dimensional (2D) cell cultures provide a simple, inexpensive, and easily reproducible model, widely regarded as a first evaluation step in preclinical studies and drug discovery [1]. On the contrary, in recent years, it has been recognized that these models fall short in mimicking the original in vivo tumor architecture and also provide limited cell–cell and cell–matrix interactions compared with real-life scenarios, where tumors exhibit three-dimensional (3D) growth [2,3].

The extracellular matrix (ECM), a dynamic intercellular network, in addition to its structural functions, also plays a crucial role in regulating several cellular processes under both physiological and pathological conditions, through multiple inside-out or outside-in signals [4,5]. The aberrant crosslinking of key matrix proteins and collagen accumulation leads to increased stiffness in solid tumors. Matrix stiffness, primarily mediated by enzymes such as lysyl oxidases, alters tumor cell behavior and phenotype by affecting different perspectives of cell functional properties within the TME. Two-dimensional models have previously supported the correlation between tumor matrix-derived stiffness and cancer cell behavior, whereas in 3D conditions the modification of matrix properties seems to have different responses on cancer phenotypes [6,7,8]. Moreover, it has been shown that in 3D cultures of the triple negative breast cancer cell line MDA-MB-231, cells adapt their characteristics through interactions with major ECM components such as collagen type I and Matrigel^®^ as a means of survival in different microenvironments [9]. Thus, to better understand TMEs and develop a more efficient method of anticancer drug evaluation, preclinical models require continuous evolution. Among different approaches, the creation of 3D cell culture models that simulate the growth and initial progression of solid tumors represents a promising research approach [10]. The different techniques for creating these types of platforms can be generally classified as matrix-based and matrix-independent. In matrix-based models, hydrogel- or polymer-based bioscaffolds of natural or synthetic origin are utilized to mimic the natural microenvironment of the cells and as a result, can influence their characteristics. Matrix-independent models involve the self-assembling of the cells through specialized culture techniques, like the hanging drop assay or ultra-low adhesion plates, which produce cell structures defined as spheroids. The latter enable dynamic cell–cell interactions and better mimic the physical properties of cells in vivo, which is valuable for applications like drug testing [11,12].

In recent years, breast cancer remains the leading cause of cancer death in women globally, with approximately 2.3 million new cases reported annually [13]. Due to its heterogeneity, advanced preclinical models that closely mimic the TME are a requirement for the evaluation of new therapeutic agents, which is a primary focus of current work. This review highlights the need to adopt spheroid breast cancer models and aims to provide improved preclinical drug evaluation, contributing to the discovery of novel therapeutic targets.

## 2. Spheroids as Effective Models That Replicate the Structural and Functional Characteristics of Solid Tumors

**Figure 1:**
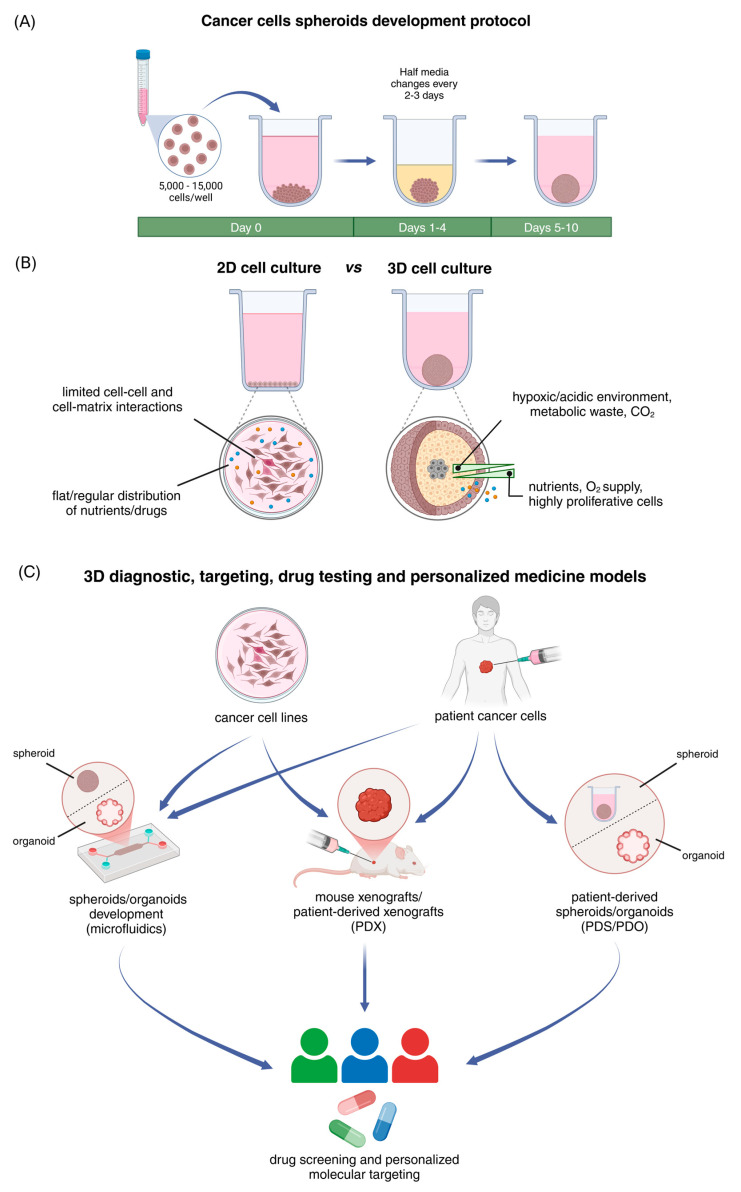
Development and applications of 3D cancer cell-derived spheroids and comparison of 2D vs. 3D cell cultures. (**A**) Three-dimensional cancer cell-derived spheroids development protocol. Cancer cells are seeded in U-shaped, round bottom plates with ultra-low adhesive properties at a density of 5000–15,000, depending on the experimental requirements. Cells are incubated for 72 h in complete medium without any medium change at 37 °C in an atmosphere of 5% CO_2_ and 95% air, allowing for spheroid development. After that, half medium changes are conducted every 2–3 days, supporting further spheroid growth. (**B**) Major differences between 2D and 3D cell culture models. Traditional 2D cell cultures comprise of cells grown in monolayers, exhibiting limited cell–cell and cell–matrix interactions and uniform exposure to nutrients, O_2_, and drugs. Because of these limitations, research has shifted to the development of advanced 3D cell culture systems. Three-dimensional cell models more accurately replicate the dynamic cell–cell and cell–matrix interactions found in solid tumors in vivo. The cellular heterogeneity found in the 3D spheroids creates gradients of nutrients, O_2_/CO_2_, and pH, as well as differential drug exposure in the distinct cell layers. (**C**) Three-dimensional preclinical models for diagnosis, drug screening, and personalized medicine. Cancer cell lines or patient-derived cancer cells can be utilized to generate tumor spheroids or organoids, using microfluidic devices or mouse xenografts. These 3D models offer a more representative platform for drug testing, advancing personalized medicine, and precision molecular targeting. Created in BioRender.com, accessed on 25 February 2025.

Three-dimensional cell culture platforms have emerged as a promising approach, bridging the gap between traditional cell cultures and animal models in preclinical studies [14]. Importantly, the development and application of innovative in vitro 3D cellular models are crucial for unraveling the complex dynamics of cancer biology and translational cancer research [15]. Among various 3D platforms, tumor spheroids represent a simple yet advanced model that effectively mimics the structural and functional characteristics of in vivo solid tumors [14]. By incorporating both cell–cell and cell–matrix interactions, they provide a powerful and more representative platform for studying the TME properties [16]. Spheroids exhibit topography, metabolism, signaling, and gene expression levels that closely resemble those of cancer cells in multilayered in vivo solid tumors [17]. Regarding their spatial organization, spheroids consist of three distinct cellular zones: (a) an outer layer consisting of highly proliferative cells, (b) an intermediate layer containing quiescent, less metabolic cells and (c) an inner core, characterized by hypoxic and acidic conditions [18]. This cellular heterogeneity creates critical gradients of nutrients and signaling molecules, O_2_ or CO_2_, pH, and drug penetration, properties that make spheroids an invaluable tool for tumor progression and drug resistance studies (Figure 1B) [19].

Recognizing the ECM as a crucial component of the TME and its pivotal role in cancer progression, platforms for spheroid development are classified into matrix-based and matrix-independent systems, based on whether or not bioscaffolds are used to embed the 3D cell culture models [12,20]. Matrix-based platforms offer a 3D artificial microenvironment that is similar to native tissues, allowing for dynamic cell-cell and cell-matrix interactions within spheroids [12,21]. Importantly, the physicochemical and biomechanical features of the utilized bioscaffolds drive the morphology, signaling, growth, and functional properties of cancer cells [11,12,22]. Various biomaterials can be utilized in these scaffolds (for further details, refer to [23]) [12,24,25,26,27,28].

Three-dimensional cell cultures in scaffold-free platforms are able to deposit their own ECM, thereby also developing intricate cell-to-cell and cell-to-matrix interactions [24,28]. Interestingly, studies show that the de novo matrix deposition is generated in a cell line- and culture-dependent manner [24]. Liquid overlay, hanging drop, spinner culture, and magnetic levitation are among the most commonly used techniques for spheroid formation, all of which promote cell-cell adhesion and facilitate cell aggregation [29,30]. Matrix-independent techniques are widely utilized due to their simplicity and minimum laboratory requirements, low cost, high reproducibility, and suitability for high-throughput drug screening, while they also offer the opportunity for co-cultures [11,28,29]. Focusing on the scaffold-free liquid overlay technique, which relies on culture plates with ultra-low adhesive properties, we provide the experimental protocol (based on conventional spheroid culture models) followed by our research group for generating 3D cancer cell-derived spheroids (Figure 1A).

Three-dimensional cancer models, such as spheroids (and organoids), are emerging as advanced preclinical tools that not only enhance cancer research, but also pave the way for precision medicine and targeted therapies (Figure 1C) [31,32]. Of note, microfluidic devices have become valuable platforms for spheroid/organoid development, allowing for cancer modeling and efficient drug screening [33,34,35]. Currently, cancer patients with similar types of cancer usually receive similar treatments regardless of their genetic profile [36]. To this regard, patient-derived spheroids/organoids (PDS/PDO), as well as patient-derived xenografts (PDX), provide essential platforms for personalized diagnosis and high-throughput drug screening [36,37].

Spheroids exhibit cellular interactions that more closely resemble expression profiles found in in vivo conditions [38,39,40]. Particularly, gene expression analyses have indicated similarities in the number of transcripts between the 3D models and in vivo groups in comparison with the respective 2D cultures [41,42]. Numerous research groups have demonstrated variations in gene and/or protein expression levels associated with cancer progression, as well as differential drug response, between 2D monolayers and 3D platforms (Table 1) [43]. For instance, Abbas et al. revealed significant alterations in the expression of genes implicated in colorectal cancer progression (matrix-independent platform), affecting properties such as proliferation, hypoxia, cell adhesion, and stemness characteristics [1]. Gene expression profiles were also substantially different between lung cancer cells cultured in 2D monolayers and those grown in 3D conditions, embedded in Matrigel^®^. Espinoza et al. reported an upregulation of genes associated with lung cancer progression in 3D models, particularly those involved in hypoxia signaling, epithelial-to-mesenchymal transition (EMT), and tumor microenvironment regulation [44]. Significant alterations in the gene and protein expression levels of EMT markers were also demonstrated by Jang et al. in gastric cell microtumors, cultured as cell-laden collagen beads within a microfluidic device [45].

Additionally, 3D patient-derived head and neck squamous cell carcinoma spheroids showed differential protein expression profiles of epidermal growth factor receptor (EGFR), EMT, and stemness markers. Interestingly, patient-derived cells grown in 3D conditions—in ultra-low adhesive plates—demonstrated greater viability following treatment with escalating doses of cisplatin and cetuximab [46]. Higher EGFR expression was also noted in 3D cultured pancreatic ductal adenocarcinoma cells compared to the respective 2D cells [47]. Koedoot et al. further identified a unique gene expression profile in breast cancer cells cultured in a 3D bioscaffold composed of Matrigel^®^ and collagen, compared to 2D cultures. Their results indicated significant alterations in the expression of genes implicated in breast cancer progression and metastasis, particularly cell cycle regulators and matrix organization molecules, while a differential response to inhibitors was also noted [42]. Moreover, 3D patient-derived cervical cancer spheroids—cultured in ultra-low adhesive plates—exhibited significantly higher expressions of genes associated with hypoxia, angiogenesis, immune responses, and matrix remodeling [48]. Finally, regarding drug response, several research groups have observed variations in the expression of drug resistance genes and proteins between 2D and 3D cell models among different types of tumors, including lung, prostate, renal, and breast cancer, as well as melanoma, lymphoma, and hepatocellular carcinoma [49,50,51,52,53,54]. These representative findings collectively underscore the impact of 3D culture systems in better recapitulating the in vivo TME complex interactions, highlighting their relevance in studying cancer progression, gene expression dynamics, and therapeutic responses across various tumor models (Table 1).

**Table 1:** Differential gene and/or protein expression levels associated with cancer progression properties and ECM composition in various tumors in 2D monolayers vs. 3D cell models.

Cancer Properties/ECM Composition	Differential Gene/Protein Expression in 2D vs. 3D Cell Models	Cancer Type	References
Proliferation/growth	*AURKA*, *AURKB*, *CDK1*, *CDK2*, *CDK4*, *CDK5*, *CDK8*, *CDK16*	Breast cancer	[42]
	c-Myc, Rac1	Lymphoma	[53]
	*PIM2*	Neuroblastoma cancer	[55]
	MYC	Colorectal cancer	[56]
	MYC	Lung cancer	[44]
EMT/MET	CDH1 *, CDH2 *, VIM *, *FN1*, *TWIST1*	HNSCC	[46]
	*TWIST1*, *CDH2*, *VIM*, *FN1*	Lung cancer (NSCLC)	[49]
	CDH1 *, VIM *, Claudin, N-cadherin	Breast cancer	[57,58]
	*EPCAM*, *VIM*	Lung cancer	[44]
	*FN1*	Ovarian/Cervical cancer	[48,59]
	Vimentin, P-Cadherin, N-Cadherin, E-Cadherin, *β*-catenin, Snail	Ovarian cancer	[60,61]
	E-cadherin, *β*-catenin, Vimentin, N-Cadherin, Fibronectin	Gastric cancer	[45]
	CDH1 *, CDH2 *, VIM *, SNAI1 *, SNAI2 *, TWIST1 *, ZEB1 *	Prostate cancer	[62]
Migration/invasion	*MMP2*, *MMP9*	Lung cancer (NSCLC)	[49]
	*MMP1*	Ovarian/Cervical cancer	[48,59]
	MMP-2, MMP-9, Tiam1	Lymphoma	[53]
	MMP-9	Colorectal cancer	[63]
	*MMP9*, *MMP14*	Lung cancer	[44]
Receptors	*CD44*	Colorectal cancer	[1]
	CD44 isoforms	Prostate cancer	[62]
	EGFR	HNSCC	[46]
	EGFR, HER2, HER3, HER4	Breast cancer	[52]
	EGFR	Pancreas cancer	[47]
	*ESR1*, *ESR2*	Thyroid cancer	[64]
Stemness	*OCT4*, *SOX2*	Colorectal cancer	[1]
	*NANOG*, SOX2 *	HNSCC	[46]
	*NANOG*, *SOX2*, *CD133*, *POU5F1*, *ALDH1A3*	Lung cancer (NSCLC)	[49]
	*ALDH1A1*, *SERPINB3*, *SERPINB5*, *CDH3*, *KRT19*	Breast cancer	[42]
	c-Kit, Sca-1	Lymphoma	[53]
	*NANOG*, *SOX2*, *POU5F1*	Thyroid cancer	[64]
	*ALDH3A1*	Melanoma, renal cancer	[51]
	CD117, CD133	Prostate cancer	[65]
ECM composition/deposition	*P3H3*, *P3H4*, *PLOD1*, *PLOD3*, *TIMP2*, *ITGB1*, *LAMA3*, *LAMA4*, *COL4A1*, *COL4A2*	Breast cancer	[42]
	*LAMC2*	Ovarian cancer	[59]
	*COL1A1*	Lung cancer	[44]
Drug response/Drug resistance	*MDR1*, *ABCG2*	Lung cancer (NSCLC)	[49]
	*MRP1*, *LRP*	Prostate cancer	[50]
	MDR1, MRP1, BCRP	Lymphoma	[53]
	MDR1 *	Melanoma, renal cancer	[51]
	BCRP	Breast cancer	[52]
	TP53	Hepatocellular carcinoma	[54]

* Also indicated in the protein level. NSCLC, non-small cell lung cancer; HNSCC, head and neck squamous cell carcinoma.

## 3. Three-Dimensional Spheroids—Models in Studying Tumor Development and the Initial Stages of Tumor Spreading/Cancer Cell Dissemination

Tumor spheroids comprise a valuable in vitro model for studying tumor growth and cancer progression, with multicellular tumor spheroids exhibiting growth kinetics comparable to those of in vivo tumors [21,66]. By closely mimicking the native tumor architecture, they offer a robust platform for exploring the intricate crosstalk between cancer cells, the tumor mass, and the TME during cancer development and progression [67]. Of note, 3D spheroids serve as a powerful tool particularly for studying avascular tumors, where the characteristics of the avascular TME significantly influence tumor development [68,69]. All tumors undergo an avascular growth stage, during which nutrients are supplied by the surrounding tissue. This stage can be more efficiently investigated through both qualitative and quantitative experiments using tumor spheroids in vitro [70].

Cancer metastasis is the leading cause of cancer-related mortality, responsible for nearly 90% of cases. While survival rates have improved through early detection and tumor growth control, effective treatments for metastasis remain limited and challenges in detecting metastatic dissemination before overt metastases develop still persist [71,72]. Metastasis begins with invasion, where tumor cells break the basement membrane and migrate through the ECM into surrounding tissues, initiating their spreading to distant organs [73]. EMT plays a crucial role in this process by increasing cancer cell plasticity. This transition allows cells to shift from a stationary epithelial state to a motile mesenchymal phenotype, enhancing their ability to migrate and invade, while also resisting apoptosis and treatment [74]. In vitro models for studying cancer cell migration and invasion primarily include trans-well migration assays, wound healing assays, and time-lapse cell tracking in 2D monolayer cultures, to assess cellular motility; however, these models fail to accurately capture the EMT process. As a result, recent trends have shifted towards 3D models of invasion [75,76].

Importantly, tumor spheroids serve as more representative in vitro models for investigating metastasis than 2D monolayers [68,75]. Kunjithapatham et al. have demonstrated that human hepatocellular carcinoma cell-derived spheroids retain their malignant characteristics, including EMT, cancer stemness, and metastatic potential after reverting to 2D monolayer cultures [77]. Coelho et al. further revealed that breast cancer cell-derived spheroids replicated key characteristics of in vivo metastasis, such as collective cell migration across the periphery of spheroids and induction of EMT process, as evidenced by changes in protein expression levels [75]. Moreover, Roper et al. effectively utilized medulloblastoma cell-derived spheroids as a model to study the initial stages of metastatic dissemination, providing significant insights into key changes occurring in migratory cells [78]. Importantly, several protocols have been described by Nazari for generating breast cancer cell-derived spheroids with a thin sheet of basement membrane to model tumor growth, as well as their embedding into a collagen-rich matrix scaffold for studying the initial invasion of mammary epithelial cells [79].

**Figure 2:**
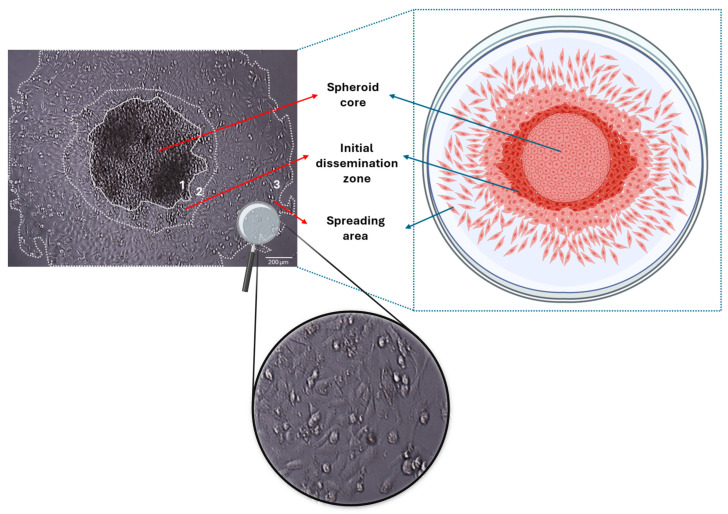
Dissemination of cancer cell-derived spheroids as a model to mimic the initial stages of tumor spreading. The aggressive, triple negative breast cancer cell line MDA-MB-231 cells were cultured for 72 h in ultra-low adhesion plates (allowing for spheroid development) and then transferred to a flat-bottom (2D) polystyrene well plate. The representative microscope image on the left panel was captured 48 h after transitioning from 3D to 2D culture (scale bar, 200 μm). Interestingly, as shown in the cartoon on the right panel, cells appear in three distinct zones. In the spheroid core zone (1), cells remain densely packed and high in number. In the initial dissemination zone (2), cells appear broken away from the spheroid core but remain close to it, exhibiting a more globular shape. In the spreading area (3), cells seem to acquire their 2D morphology (mesenchymal-like phenotype, as noted for 2D MDA-MB-231 cells) as shown in the bottom panel in greater magnification. Created with BioRender.com.

Given the notable differences in gene and protein expression profiles between 2D and 3D cell culture models, we further aimed to highlight the potential of our 3D spheroid model as a more relevant platform for exploring the initial stages of cancer cell dissemination. As shown in Figure 2, MDA-MB-231 cells are spreading from the entire periphery of the spheroids after being transferred to standard flat-bottom plates.

## 4. Conclusions and Future Perspectives

In recent years, advancements in 3D cell culture models have enhanced our understanding of cancer cell behavior and function. Among these, spheroids bridge the gap between conventional 2D cell cultures and in vivo models, unraveling a new era in cancer research. Particularly, spheroids can aid in the testing of potential treatments through the evaluation of drug penetration and accumulation [80]. Spheroid characterization is an essential step in cancer research to establish a reliable model [81]. Recently, deep learning has emerged as a powerful tool for assessing spheroid viability in a label-free manner, offering a high-throughput alternative to conventional biochemical assays, which require chemical or physical stimuli. By leveraging microscopic image-based analysis, deep learning models can provide accurate and real-time viability assessments, improving the efficiency of drug screening studies [82,83].

Despite their potential, spheroids generated from cancer cell lines face some challenges. Cancer cell lines do not accurately represent all patients, as each individual possesses a unique molecular profile. In addition, over time, cancer cell lines can also develop genetic and epigenetic alterations that may diverge from the original tumor’s traits, potentially influencing the outcomes of spheroid model experiments [28]. Organoids have emerged as a 3D model capable of addressing this challenge. Derived from stem cells, organoids consist of organ-specific cell types that self-organize through cell sorting and spatially restricted lineage commitment [84]. PDO are particularly valuable as they preserve primary tumor heterogeneity, making them ideal for biomarker identification and drug response testing. However, their high production cost and the lack of key tissue components, such as vascular and immune cells, limit their ability to replicate cell–matrix and cell–stroma interactions completely [11].

Furthermore, the use of 3D tumor models holds a great future in advancing cancer research, as they are essential for testing new anticancer drugs and therapies. However, integrating personalized medicine through patient-derived organoids raises ethical concerns, particularly regarding the source of stem cells, informed consent, and issues of equity in access to such treatments. Additionally, questions surrounding organoid biobanking, potential commercialization, and the affordability of personalized therapies highlight the need for regulatory frameworks to ensure ethical and fair application [36]. Ultimately, the use of patient-derived tumor spheroids can facilitate personalized drug screening and treatment selection, bridging the gap between laboratory research and clinical application and thus providing new insights for developing novel anticancer therapies.

## Abbreviations

The following abbreviations are used in this manuscript:

2D/3D	Two-dimensional/three-dimensional
ECM	Extracellular matrix
EGFR	Epidermal growth factor receptor
EMT	Epithelial-to-mesenchymal transition
PDO	Patient-derived organoids
PDS	Patient-derived spheroids
PDX	Patient-derived xenografts
TME	Tumor microenvironment
